# Evaluating ethanol concentrations against *Staphylococcu*s spp: a proposal for improving nosocomial bacteria control

**DOI:** 10.1016/j.infpip.2023.100336

**Published:** 2023-12-27

**Authors:** Ferda Soyer, Ozgun Oyku Ozdemir, Bengi Polat, Nil Hazal Ekenel

**Affiliations:** a Department of Molecular Biology and Genetics, Izmir Institute of Technology, Izmir, Turkey; b Department of Pharmaceutical Microbiology, Faculty of Pharmacy, Kocaeli Health and Technology University, Kocaeli, Turkey

Dear Editor,

Nosocomial infections originating from commonly encountered pathogenic bacteria, notably *Staphylococcus* species, persist as a prominent global public health issue. This phenomenon exerts consequential impacts on both the well-being of patients and the healthcare personnel within hospital environments. Hospital-acquired infections from common bacteria like *Staphylococcus* remain a global public health concern. The European Centre for Disease Prevention and Control reports prevalence rates of 4.5% in the USA and 7.1% in Europe [1]. An estimated 8.9 million healthcare-associated infections occur annually in European hospitals and long-term care facilities [1]. According to the World Health Organization, although 10% of patients get healthcare-associated infections, at least a 30% reduction can be achieved through adequate infection prevention and control [2]. The efficacy of disinfection methodologies employed in healthcare institutions assumes critical significance in mitigating this threat.

Antibiotic-resistant microorganisms, notably methicillin-resistant *Staphylococcus aureus* (MRSA), prolong hospital stays and increase mortality risk, contributing to 90,000 annual deaths in the US alone [3]. Staphylococci, especially *Staphylococcus aureus* (*S. aureus*) and *Staphylococcus epidermidis* (*S. epidermidis*), play a significant role in hospital-acquired infections, including those linked to medical devices [4]. Hospital surfaces sustain MRSA for extended periods, facilitating transmission. Despite widespread hospital use, our study reveals that 70% ethanol fails to eliminate *Staphylococcus* species, potentially perpetuating nosocomial infections. Absolute ethanol is impractical due to rapid evaporation and bacterial adhesion. Ethanol concentrations between 60-95% are generally effective, with 80–85% considered optimal [5]. Higher concentrations exhibit reduced potency, while concentrations below 50% are less effective. Ethanol-based hand rubs with concentrations between >75% v/v and <95% v/v, applied for at least 3 minutes, demonstrate bactericidal efficacy comparable to the reference procedure in meeting EN 12791 requirements for hand hygiene, as determined through comparative testing with n-propanol [6]. Fifteen seconds of exposure to 85% ethanol significantly reduces bacterial load [7]. Standard commercial formulations with 63% ethanol may inadequately combat *S. aureus* [8]. According to WHO guidelines, dryness within 10–15 seconds post-hand rubbing indicates insufficient product application [9]. Comparing 70% and 80% ethanol evaporation times on hand, applying 1 mL of each solution revealed a 4-second average decrease with 80% ethanol, constituting a 15% reduction in evaporation time (Soyer Lab, unpublished data). We recommend using ethanol at 80–85% concentrations in healthcare settings for enhanced efficacy, resembling the impact of 25% household bleach. This approach may mitigate nosocomial infections, alleviating the financial burden associated with prolonged antibiotic therapy or surgical interventions.

In this study, inhibition zones were determined through the application of 15 μl volumes of various solutions (prepared in sterile ddH2O) containing 70%, 75%, 80%, and 85% ethanol (V/V), as well as 25% and 50% bleach (V/V) onto tryptic soy agar (TSA) plates previously inoculated with bacterial cultures at a concentration of 10^8^ CFU/mL using the spread plating method. Following a 15-minute incubation period at room temperature to allow absorption into the agar, the plates were incubated at 37 °C for 18 hours. All solutions were prepared fresh and used immediately in the experiment. The agar diffusion tests were independently conducted in triplicate, and the results were similar. Some of the results are depicted on the left side of Figure 1. The corresponding tables on the right side of Figure 1 provide details on the observed diameters of the inhibition zones for each solution in average millimeters with standard deviation values, along with observations regarding secondary bacterial growth (growth of non-susceptible bacteria from the same inoculation within the inhibition zone towards the end of incubation: Figure 1A. 75% Ethanol section clearly illustrates) within the inhibition zones.

**Figure 1:**
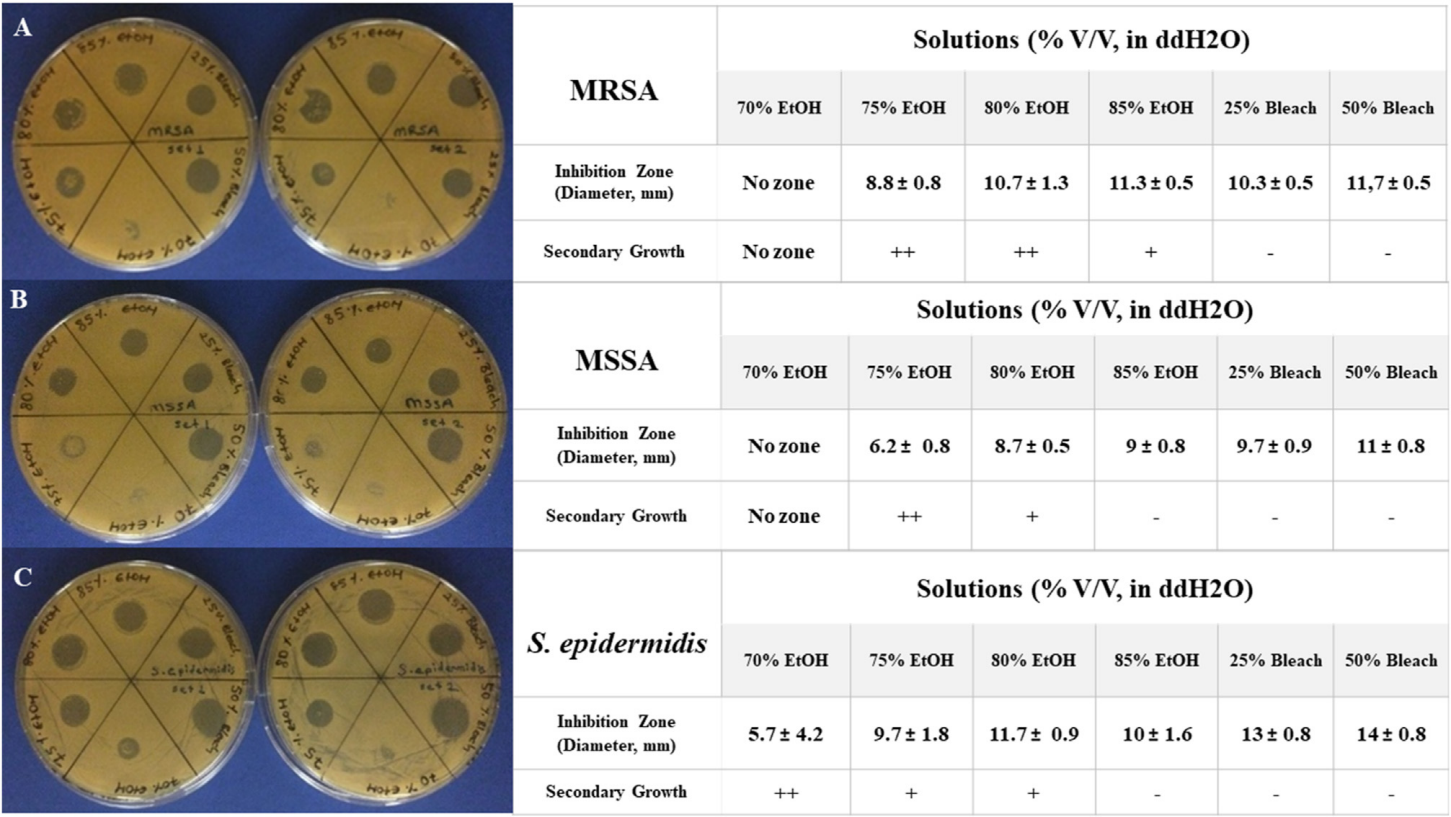
Inhibitory effects of ethanol and bleach solutions on *Staphylococcus spp.* growth. The left side showcases agar plates organized in pairs, delineating two distinct sets of bacterial cultures denoted as Set 1 on the left and Set 2 on the right for comparative analysis. On the right side of the figure, the observed diameters of the inhibition zones are quantified in millimeters for both sets of bacterial cultures, accompanied by an evaluation of secondary bacterial growth manifested within these inhibition zones. The presence or absence of secondary growth is systematically denoted by ++ and - signs, respectively. The presence of secondary growth in single colony formation within the inhibition zone is indicated explicitly with a + sign. A) MRSA B) MSSA C) *S. epidermidis*. Data represents the mean (±SD) of three plates.

MRSA exhibited extensive proliferation within the inhibition zone generated by 70% ethanol, effectively encompassing the entire area, originating from the central region of the zone. Notably, discernible single colonies of the bacteria were evident within the inhibition zones produced by 75% and 80% ethanol. Furthermore, the inhibition zones of 70% and 75% ethanol were nearly entirely obscured by the secondary growth of MSSA. The inhibitory effect of 80% ethanol resulted in the development of isolated colonies of MSSA within the respective inhibition zone. Similarly, discernible secondary growth of *S. epidermidis* manifested within the 70% ethanol inhibition zone. Additionally, single colonies of *S. epidermidis* were observed within the inhibition zones created by 75% and 80% ethanol.

The inhibitory efficacy of 70% ethanol was consistently demonstrated, as the resulting inhibition zones were nearly completely occluded by robust bacterial growth across all assessed *Staphylococcus* species. In the context of 75% ethanol, a distinct pattern emerged in the inhibition zones of both MRSA and MSSA, wherein bacterial growth manifested centrally in a ring-like configuration. This notable observation raises the possibility of secondary bacterial growth within regions subjected to the application of 75% ethanol.

Furthermore, the application of 80% ethanol revealed conspicuous and discernible inhibition zones specific to *Staphylococcus* species, as depicted in Figure 1. This distinct response underscores the varying impact of 80% ethanol concentration on the growth dynamics of the examined *Staphylococci* strains.

These results lead us to propose the usage of concentrations of 80% or 85% ethanol rather than 70% in the disinfection process in healthcare facilities for more effective elimination of MRSA, MSSA, and *S. epidermidis*. Ethanol is a highly trusted disinfectant widely used and vital in healthcare settings. We suggest that increasing the concentration of ethanol, which is used in hospitals for disinfection processes, from 70% to at least 80% (V/V; in water) would aid in better disinfection and the reduction in the spread of nosocomial bacteria, potentially preventing the deaths of thousands of people.

## Ethical statement

Not required.

## Conflict of interest statement

No conflicts of interest related to this study and letter.

## Funding

This study was funded by the 10.13039/501100003984Izmir Institute of Technology Research Grant (Project No: 2022İYTE-3-0028 to FS).
